# The Analyses of Chemical Components From *Oldenlandia hedyotidea* (DC.) Hand.-Mazz and Anticancer Effects *in vitro*

**DOI:** 10.3389/fphar.2021.624296

**Published:** 2021-05-10

**Authors:** Chuanyi Zhao, Mengyuan Wei, Yilin Zheng, Weili Tao, Qian Lv, Qiongjin Wang, Shuyun Wang, Yicun Chen

**Affiliations:** Department of Pharmacology, Shantou University Medical College, Shantou University, Shantou, China

**Keywords:** Oldenlandia hedyotidea (DC.) Hand.-Mazz, UHPLC-MS, UHPLC-Triple-TOF-MS, fingerprint, proliferation, migration

## Abstract

*Oldenlandia hedyotidea* (DC.) Hand.-Mazz (OH), also known as sweet tea, is a valuable functional food with medicinal properties and is used for the treatment of cold, cough, gastroenteritis, heatstroke, herpes zoster, and rheumatoid arthritis. The phytochemicals in plant-based foods are responsible for the occurrence of these diseases to some extent. However, there is a scarcity of information on the chemical components of OH. We, therefore, aimed to investigate the phytochemical components of OH using ultra high-performance liquid chromatography–mass spectrometry (UHPLC-MS) and UHPLC triple time-of-flight mass spectrometry (UHPLC-Triple-TOF-MS). The main component of the OH extract, asperulosidic acid, was additionally quantified using UHPLC with ultraviolet detection (UHPLC-UV). The anticancer activity of the OH extract was assessed by a cell proliferation assay and a scratch assay using an esophageal cancer cell line. Ten compounds were tentatively identified in the aqueous extract of OH, including five iridoids, two anthraquinones, and one phenolic acid. The content of asperulosidic acid in the aqueous extract of OH was approximately 42 μg ml^−1^, and the extract exerted definite *in vitro* anticancer effects. The results can be used for quality control and assessment of the OH extract, which can serve as a promising source of functional ingredients for potential use in the food and drug industries.

## Introduction

*Oldenlandia hedyotidea* (DC.) Hand.-Mazz, (syn. *Hedyotis hedyotidea* (DC.) Merr., Niubaiteng in Chinese; Rubiaceae is cultivated in the south of China, including the Guangdong, Guangxi, Yunnan, Fujian, and Taiwan provinces, and yields a herbal tea. It is used for the treatment of cold, cough, gastroenteritis, heatstroke, herpes zoster, and rheumatoid arthritis (Peng et al., 1998). It is also used in several prescribed herbal formulations, including Niu-Bai-Teng-He-Si-Miao-Tang (He et al., 2012b) and Fu-Fang-Niu-Bai-Teng-Tang (Wei, 1978). It is also used to prepare herbal tea by the people living in the southern provinces, who consider it a healthy drink for preventing cold and treating cough. It is hypothesized that these effects are attributed to the chemical components of sweet tea; however, few studies have reported the chemical composition and quality control of OH.

Related species with antitumor activity include *Scleromitrion diffusum* (Willd.) R. J. Wang (SD) (also named *H. diffusa Willd*, Baihuasheshecao in Chinese), which has been clinically used for the treatment of lung cancer (Lin et al., 2019), liver cancer (Chen et al., 2012; Li et al., 2016), malignant melanoma (Ling et al., 2016), ovarian cancer (Zhang et al., 2016), and especially colorectal cancer (Yan et al., 2017; Li Q. et al., 2018; Song et al., 2019), which is a type of gastrointestinal cancer. OH is used for the treatment of leukemia in the Chaoshan area of the Guangdong Province (Cheng, 2013). However, there are no reports on whether OH is effective in treating gastrointestinal cancers. Esophageal cancer (EC) is one of the most common types of gastrointestinal cancers. Esophageal squamous cell carcinoma (ESCC) is the most frequent subtype of EC and is highly prevalent in China, especially in the Chaoshan region (Su et al., 2007; Torre et al., 2015). The human ESCC cell lines include EC109, KYSE140, KYSE410, and KYSE510. In this study, we determined the anticancer activity of the aqueous extract of OH by a cell proliferation assay and a scratch assay using an esophageal cancer cell line.

In this study, we attempted to detect and characterize the major constituents in OH using ultra high performance liquid chromatography–mass spectrometry (UHPLC-MS) (Li et al., 2003; Li J. et al., 2018; Siegle and Pietsch, 2018) and UHPLC triple time-of-flight mass spectrometry (UHPLC-Triple-TOF-MS) (Zhao et al., 2017; Huo et al., 2018), for identifying the major constituents of OH for the first time. By combining three identification techniques, namely, plant DNA barcoding (Chen et al., 2010; Yao et al., 2010), thin-layer chromatography (TLC) (Mustarichie et al., 2019; Ansari et al., 2020; Jakinala et al., 2021), and microscopic identification (Li et al., 2015), we verified the different batches of OH, both authentic and inauthentic, collected from different provinces. The results of our study are provided in the supplementary section (refer to Supplementary Figures S1, S2, S3, and S4 and Supplementary Table S1). We identified and tentatively characterized 10 components, including five iridoids, two anthraquinones, and one phenolic acid. This study is the first to report these 10 compounds in the aqueous extract of OH, with the exception of asperulosidic acid. Chromatographic fingerprinting is recognized as a convenient and efficient technology for quality evaluation and quality control (Yang et al., 2008; Sun et al., 2014) because it can provide a comparative and comprehensive account of the contents of the chemicals in complex solution systems, such as raw materials. We also determined the chromatogram fingerprints and generated a phylogenetic tree for separately analyzing the 19 different batches of OH. The chromatogram fingerprints revealed that asperulosidic acid was the main component in the aqueous extract of OH. Asperulosidic acid was subsequently quantified by UHPLC-UV, and the UHPLC-UV method developed herein was validated by measuring the accuracy, precision, specificity, linearity, and range according to the Chinese Pharmacopoeia (CH.P), 2015, chapter 9101 (similar to USP<1225>).

## Materials and Methods

### Standards and Chemicals

The reference standard of asperulosidic acid (HPLC ≥98%) and ferulic acid (HPLC ≥98%) were obtained from Shanghai Yuan Ye Biotechnology Co., Ltd (Shanghai, China). The HPLC-grade acetonitrile was purchased from Merck Drugs & Biotechnology (Darmstadt, Germany. CAS-NO: 75-05-8). The HPLC-grade formic acid was obtained from Thermo Fisher Scientific (CAS 64-18-6). Ultrapure water was obtained using a Milli-Q system (Millipore, Bedford, MA, United States). Cisplatin (CAS-NO: 15663-27-1) and 5-fluorouracil (CAS-NO: 51-21-8) were purchased from SHANTOU CENTRAL HOSPITAL. Methanol is HPLC-grade, purchased from Merck, Germany. (CAS-NO: 67-56-1).

### Plant Materials

Nineteen batches of dry OH were collected from 4 different provinces in China. All samples were identified by Zihe Luo, the professor of SHANTOU CENTRAL HOSPITAL, and deposited (YGZ 002) in the herbarium of Shantou University. They were labeled as Table 1 **.**

**TABLE 1:** Sample information of OH.

Number	Code	Source	Collection time	Medicinal part	Coordinates
1	S1	Shantou, Gangdong	20180926	Stems	116 70′E, 23 37′N
2	S2	Chaozhou, Gangdong	20180925	Stems	116 63′E, 23 66′N
3	S3	Yulin, Guangxi	20180927	Stems	110 14′E, 22 64′N
4	S4	Lijiang, Yunnan	20180927	Stems	100 25′E, 26 86′N
5	S5	Yulin, Guangxi	201806	Stems	110 14′E, 22 64′N
6	S6	Yulin, Guangxi	201807	Stems	110 14′E, 22 64′N
7	S7	Yulin, Guangxi	201808	Stems	110 14′E, 22 64′N
8	S8	Yulin, Guangxi	20180925	Stems	110 14′E, 22 64′N
9	S9	Hezhou, Guangxi	20180930	Stems	111 55′E, 24 41′N
10	S10	Hezhou, Guangxi	20180930	Stems	111 55′E, 24 41′N
11	S11	Hezhou, Guangxi	20180930	Stems	111 55′E, 24 41′N
12	S12	Hezhou, Guangxi	20180930	Stems	111 55′E, 24 41′N
13	S13	Hezhou, Guangxi	20180930	Stems	111 55′E, 24 41′N
14	S14	Hezhou, Guangxi	20180930	Stems	111 55′E, 24 41′N
15	S15	Shantou, Guangdong	20170902	Stems	116 70′E, 23 37′N
16	S16	Maoming, Guangdong	20171209	Branches and leaves	110 91′E, 21 65′N
17	S17	Guigang, Guangxi	20171213	Branches and leaves	109 60′E, 23 09′N
18	S18	Yulin, Guangxi	20171202	Stems	110 14′E, 22 64′N
19	S19	Xiamen, Fujian	20171202	Branches and leaves	118 11′E, 24 49′N

### Preparation of Standard and Sample Solutions

In order to perform qualitative and quantitative analyses, standard solutions of asperulosidic acid (364 μg ml^−1^) and ferulic acid (69.2 μg ml^−1^) were prepared with methanol. OH powder (over 60 mesh sieve) was accurately weighed (about 0.5 g) and extracted with 10 ml of H_2_O using ultrasonic bath for 30 min at room temperature; centrifugal separation was performed at 4000 rpm for 15 min, then 2 ml H_2_O was added to the centrifuge tube and centrifuged at 12,000 rpm for 10 min. All samples were filtered through 0.22 μm organic membrane (Guangzhou Jet Bio-Filtration Co., Ltd.).

### UHPLC-MS Analysis

Qualitative analysis of OH was performed by an LCMS-2020 system (Shimadzu, Japan). An InfinityLab Poroshell 120 EC-C18 column of Agilent (4.6 × 100 mm, 2.2 μm) was employed on an LCMS-2020 system (Shimadzu, Japan) for chromatographic separation and MS1 scan of the samples. The column temperature was maintained at 40°C. The mobile phase was composed of water containing 0.1% formic acid (A) and acetonitrile (B) with a flow rate of 0.30 ml/min. The detection wavelength was 238 nm. The elution program was conducted as follows: 0–3 min at 5% B, 3–20 min at 5–70% B, 20–26 min at 70% B, 26–27 min at 70–5% B, and 27–30 min at 5% B. The injection volume was 5 μL (or 10 μL when using UHPLC separately to quantify the main ingredient asperulosidic acid or prepare fingerprints of 19 batches of OH). The MS1 scan range was from 50 to 1,000 m/z using tuning files as default. The analysis of LCMS-2020 was single quadrupole mass spectrometry. The following MS conditions were used: the ESI^–^mode; nebulizer gas, 3.0 L/min; dry gas, 10.0 L/min; ion source temperature, 250°C; interface temperature, 220°C; DL temperature, 250°C; and heating block temperature, 400°C. The LC-MS scan was operated with the mass range of m/z 100–1,500.

### UHPLC-Triple-TOF-MS Analysis

In order to identify the major constituents of OH. A Waters ACQUITY™ UHPLC system (Waters Corporation, United States) coupled with the Triple TOFTM 5600+ (AB SCIEX Corporation, United States), a hybrid triple quadrupole time-of-flight mass spectrometer equipped with Turbo V sources and a TurboIon spray interface was applied. Separation was done using Agilent ZORBAX-SB C18 column (4.6 × 100 mm, 1.8 µm). The column temperature was maintained at 35°C. The mobile phases were composed of water with 0.1% formic acid (A) and acetonitrile with 0.1% formic acid (B). A binary gradient elution with a flow rate of 0.8 ml min^−1^ was employed for the separation. The detection wavelength was 238 nm. The consecutive program was as follows: 5% B from 0 to 2 min, 5–50% B from 2 to 25 min, and 50–95% B from 25 to 33 min. The sample injection volume was 10 μL. The following MS conditions were used: the ESI^–^mode; nebulizer gas (Gas 1), 55 psi; heater gas (Gas 2), 55 psi; curtain gas, 35 psi; turbo spray temperature, 550°C; and ion spray voltage, −4.5 kV. First-level scanning: declustering potential (DP), −100 V; and collision energy (CE), −10 eV; The TOF MS scan was operated with the mass range of m/z 100–1500. Secondary sweep: The raw data were acquired by IDA function under the product ion mode of TOF MS. In addition, an automated calibration delivery system (CDS) was used to regulate MS to make the mass error less than 2 ppm before loading the sample. Mass resolution was greater than 30,000.

### UHPLC-UV Fingerprint Similarity Analysis and Clustering Analysis

UHPLC fingerprint analysis is currently the best choice (Siegle and Pietsch, 2018). The original fingerprint chromatograms data in format (.lcd) of 19 batches of OH were exported as AIA files (*.cdf) and calculated using professional software [Similarity Evaluation System (SES) for Chromatographic Fingerprint of Traditional Chinese Medicine, composed by the Chinese Pharmacopoeia Committee (SES, Version 2012.130,723)]. The differences in the area of common peaks of 19 samples were analyzed by IBM SPSS statistics software using the hierarchical cluster (Group connection and Cosine algorithm).

### UHPLC-UV Method Validation

The quantitative analysis was performed by an LC-30A system (Shimadzu, Japan). The wavelength used of the detector was 238 nm. The proportion of the mobile phase was the same as UHPLC-MS. The UHPLC-UV method was validated for accuracy, precision, specificity, linearity, and range according to the guidelines of the Chinese Pharmacopeia (CH.P), 2015, chapter 9101.

Accuracy of the method is calculated by a recovery test. The accurate amount of asperulosidic acid standard solutions with three different concentration levels was added to sample and three replicates of each concentration. The average recoveries were determined by the formula:

$$\text{Average recovery}\;\left(\%\right)=\frac{\left(\text{detected amount}\;-\;\text{original amount}\right)}{\text{spiked amount}}\times 100\%.$$

The precision tests were performed using six replicated injections of the same sample solution in a day.

The calibration curves of asperulosidic acid for quantitation were created by establishing a relationship between the peak area (Y) and the concentration (X, µg/ml) of the standard solution. Stored standard solution of asperulosidic acid (364 μg ml^−1^) was diluted to 7.28 μg ml^−1^, 14.56 μg ml^−1^, 29.12 μg ml^−1^, 58.24 μg ml^−1^, and 116.48 μg ml^−1^ for the UHPLC-UV test.

### Cell Lines, Medium, and Cell Culture

EC109, KYSE140, KYSE410, and KYSE510 cells were obtained from ATCC (Rockville, MD, United States) and cultured in RPMI 1640 medium, modified (HyClone) containing 10% fetal bovine serum (FBS; GIBCO), and incubated overnight at 37°Cand 5% CO_2_.

### Cell Proliferation Assay

OH powder (over 60 mesh sieve) was accurately weighed (about 10 g) and extracted with 100 ml of H_2_O. The method we used was water extraction and alcohol precipitation (Zhang et al., 2021); centrifugal separation was performed at 4000 rpm for 15 min, then we prepared the water extract of OH with a final concentration of 1 g/ml by rotary evaporation. The sample was filtered through 0.22 μm organic membrane and stored in 4°C.

The effect of the aqueous extract of OH on proliferation of EC109, KYSE140, KYSE410, and KYSE510 cells was evaluated by the Cell Counting Kit-8 (CCK8, BOSTER Biological Technology Co. ltd) assay. EC109, KYSE140, KYSE410, and KYSE510 cells in exponential growth were plated at a density of 2 × 10^5^ cells/well in 100 µL of growth medium in 96-well culture plate and incubated for 48 h with various concentration of extract (1, 5, 10, 25, 50, and 100 μg/ml) which were diluted by culture medium. At the end of the treatment intervals, 10 µL of CCK8 solution was added into each well. After 2 h of incubation in a 37°C and 5% CO_2_ incubator, the absorbance was measured in a microplate reader at a wavelength of 450 nm. The blank group (the drug sample group corresponds to the blank background group) and control group (CON) cells were maintained in an environment with 5% CO_2_ at 37°C. Positive control group 1 (PCG 1) cells were treated with 100 μg/ml cisplatin. Positive control group 2 (PCG 2), cells were treated with 100 μg/ml 5-fluorouracil.

### Scratch Assay

Esophageal cancer (EC) is one of the most common malignant tumors. The human esophageal squamous cell carcinoma (ESCC) cell lines EC109, KYSE140, KYSE410, and KYSE510 (COBIOER) were used in this experiment. The cells were plated on 6-well plates and cultured until the confluence was 90%. A 100-µL pipette tip was used to draw straight lines at the bottom of each well. The residual liquid was discarded. Serum-free medium or the extract with effective IC50 was added to continue culture after washing with PBS. Images were acquired under the microscope at 0, 10, 20, and 30 h, respectively, the scratch area was calculated by ImageJ software. The healing rate was calculated as follows:

$$\text{The healing rate}=\;\frac{\left(\text{Initial scratch area}-\text{Scratch area after culture}\right)}{\text{Initial scratch area}}\times 100\%.$$

### Statistical Analysis

One-way analysis of variance (ANOVA, Student’s t test) was used to assess the significant difference of proliferation (%) between groups (SPSS version 15.0; IBM: Chicago, IL, United States, 2006). The results were presented using the GraphPad software (GraphPad Software, CA, United States). *p* < 0.01 was considered statistically significant, and *p* < 0.001 was considered highly significant.

## Result and Discussion

### Optimization of Extraction Solvents

**FIGURE 1:**
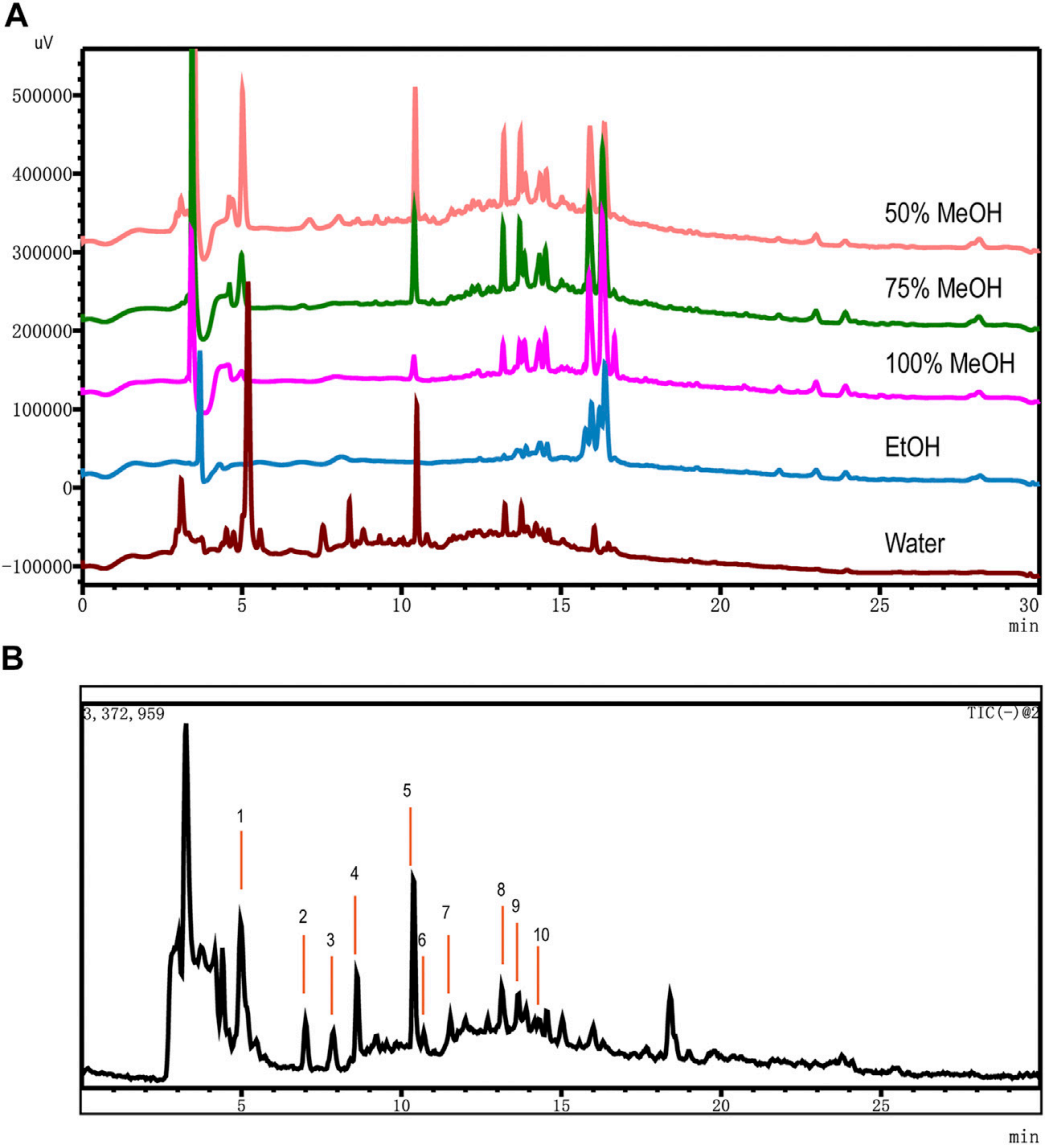
Optimization of extraction solvents and total ion chromatography (TIC) using UHPLC-MS. **(A)** Comparison of OH dissolved in different solvents: 50% MeOH (pink), 75% MeOH (green), 100% MeOH (fuchsin), EtOH (blue), and Water (brown). **(B)** TIC in the negative ionization mode of OH dissolved by water.

For acquiring an optimized extraction of OH, key factors such as solvent type methanol (50, 75, and 100%), ethanol, and water and ultrasonic time (10, 20, 30, 40, 50, and 60 min) were investigated by single variable investigation. Ultimately, the optimum sample extraction method was obtained by ultrasonic extraction at water for 30 min. The samples were all extracted at room temperature (Figure 1A).

### Optimization of UHPLC-MS Conditions

In the period of establishing analytic procedure on an LCMS-2020 system, we explored temperature (30, 40, and 50°C), different chromatographic column (InfinityLab Poroshell 120 EC-C18 column of Agilent, 4.6 × 100 mm, 2.2 μm, and Accucore C18 column of Thermo, 2.1 × 50 mm, 2.6 μm), different mobile phase (acetonitrile and methanol, LC/MS grade), and different pH (2.8 of 0.1% HCOOH, 2.9 of 0.05% HCOOH, 6.2 of 0.01 mol/L HCOONH_4_, and 8.4 of H_2_O) by comparing the data of chromatograms under different conditions visually. The ratio of signal to noise (S/N) of 10 detected compounds were greater than 3. The method described in UHPLC-MS conditions for UHPLC on the LCMS-2020 system was satisfied for the quality assessment test. MS conditions were compared in positive and negative modes, and we found that 10 components had high stability, a corresponding value, and good shape of peak in the negative ion mode (Figure 1B).

### Qualitative Analysis of OH by UHPLC-MS and UHPLC-Triple-TOF-MS

**FIGURE 2:**
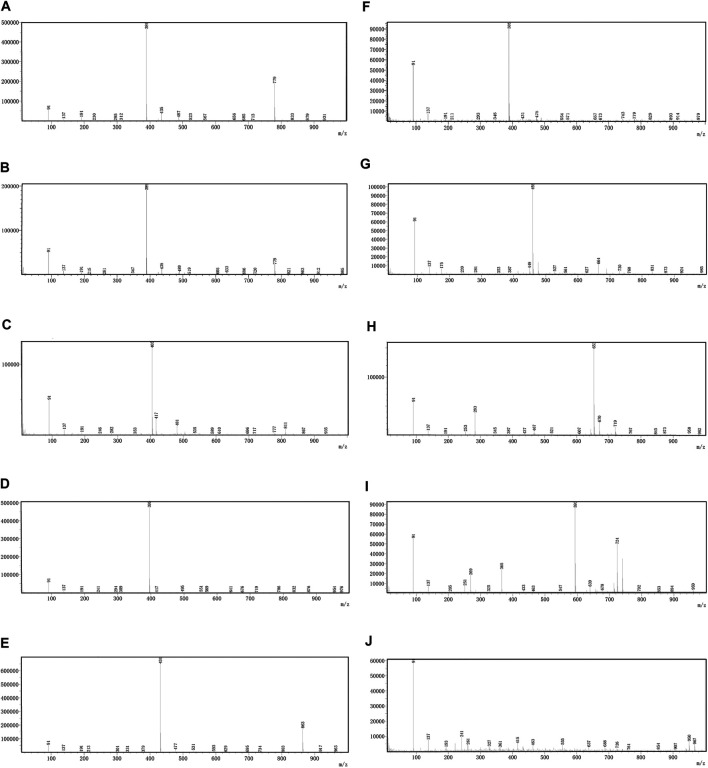
Parent ion spectrums of the main ingredients. **(A)** Monotropein. **(B)** Deacetylasperulosidic acid. **(C)** 6,7-dihydroxy-7-(hydroxymethyl)-1-(((2S,3R,4S,5S,6R)-3,4,5-trihydroxy-6-(hydroxymethyl)tetrahydro-2H-pyran-2-yl)oxy)-1,4a,7,7a-tetrahydrocyclopenta [c]pyran-4-carboxylic acid. **(D)** 3-hydroxy-4-(((2S,3R,4S,5S,6R)-3,4,5-trihydroxy-6-((sulfooxy)methyl)tetrahydro-2H-pyran-2-yl)oxy)benzoic acid. **(E)** Asperulosidic acid. **(F)** Scandoside. **(G)** Asperuloside. **(H)** Hedanthroside C. **(I)** Hedanthroside B. **(J)** Ferulic acid.

**FIGURE 3:**
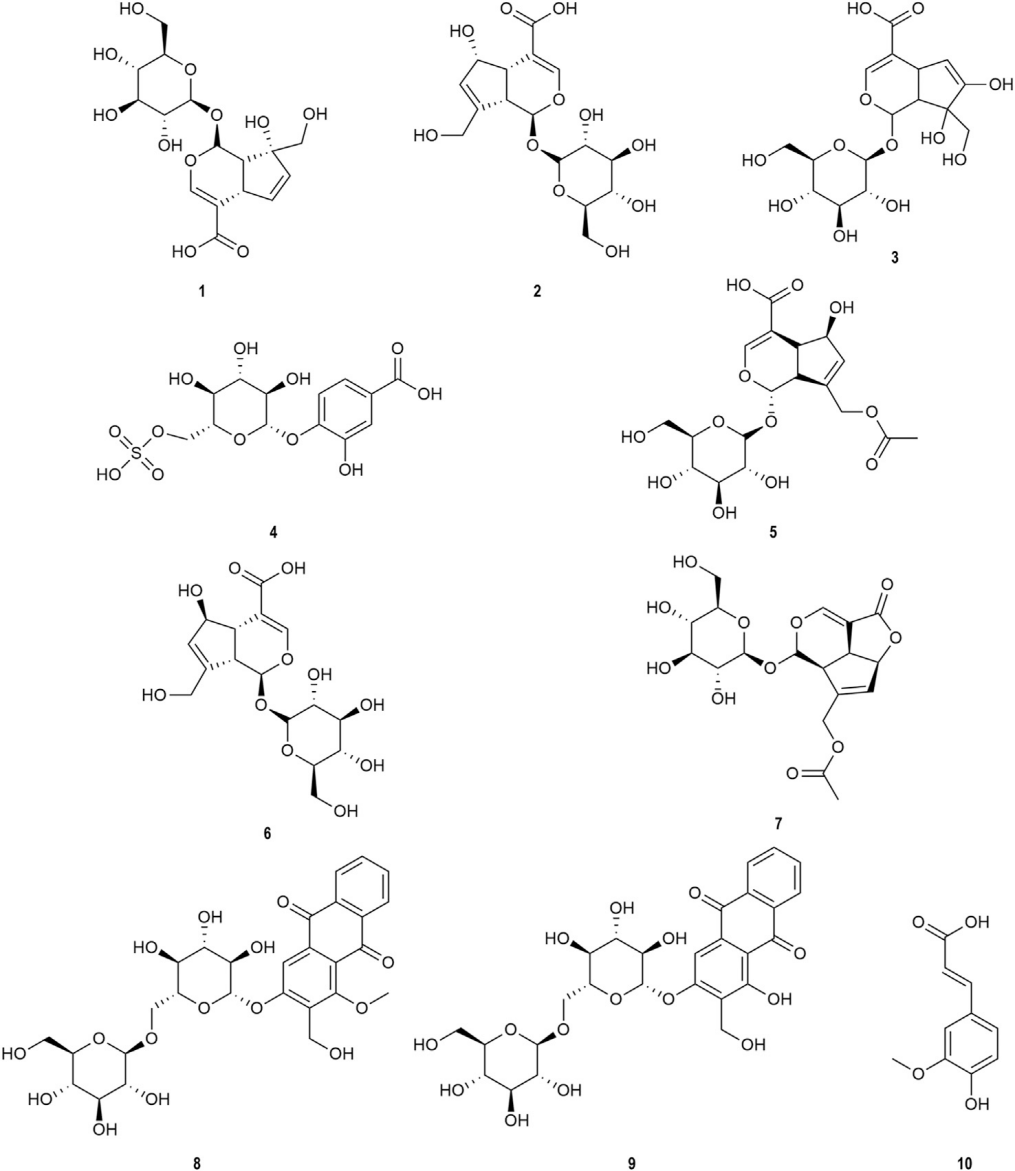
Chemical structures identified in OH. **1)** Monotropein. **2)** Deacetylasperulosidic acid. **3)** 6,7-dihydroxy-7-(hydroxymethyl)-1-(((2S,3R,4S,5S,6R)-3,4,5-trihydroxy-6-(hydroxymethyl)tetrahydro-2H-pyran-2-yl)oxy)-1,4a,7,7a-tetrahydrocyclopenta [c]pyran-4-carboxylic acid. **4)** 3-hydroxy-4-(((2S,3R,4S,5S,6R)-3,4,5-trihydroxy-6-((sulfooxy)methyl)tetrahydro-2H-pyran-2-yl)oxy)benzoic acid. **5)** Asperulosidic acid. **6)** Scandoside. **7)** Asperuloside. **8)** Hedanthroside C. **9)** Hedanthroside B. **10)** Ferulic acid.

**FIGURE 4:**
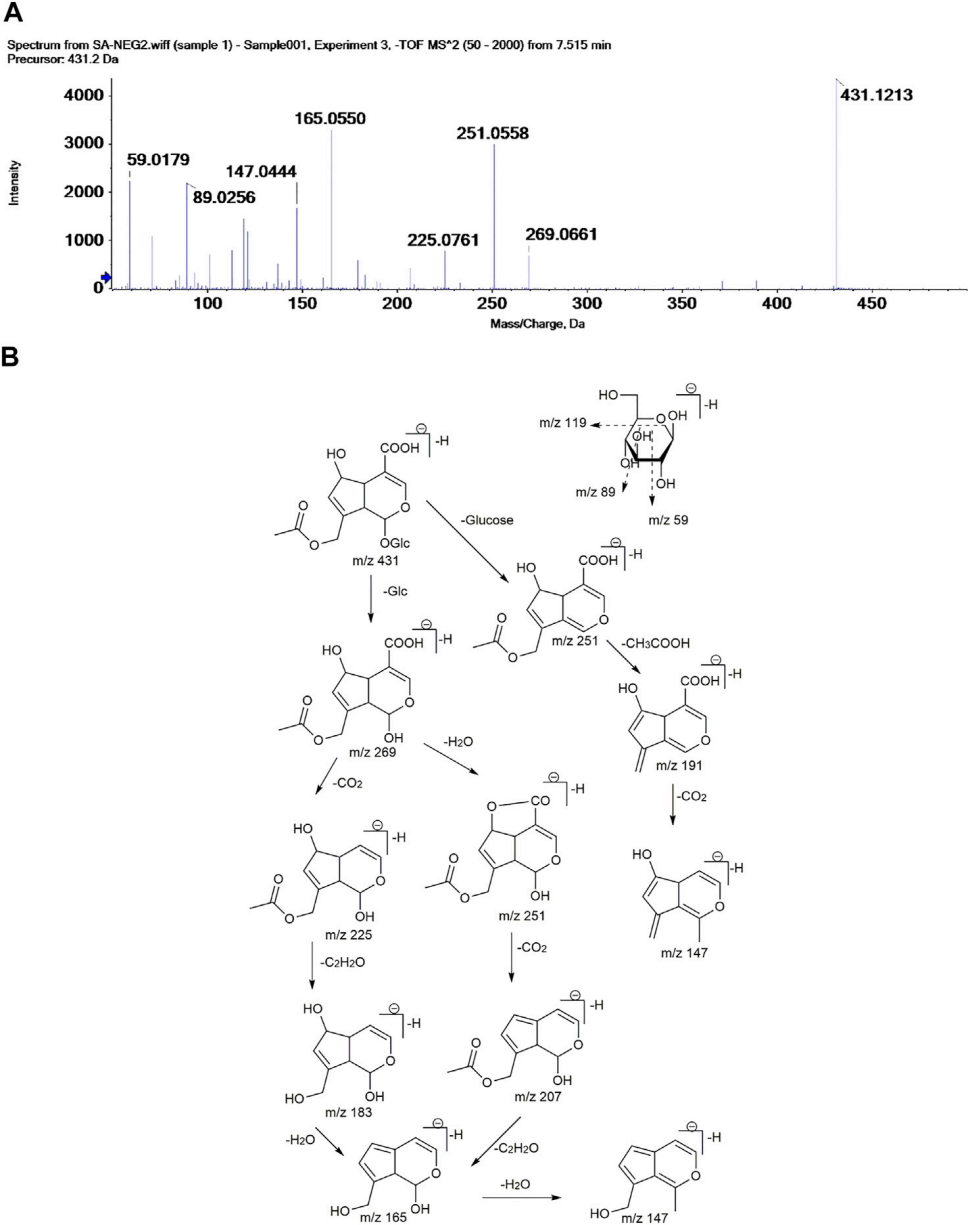
MS/MS spectra **(A)** and the proposed fragmentation pathway **(B)** of asperulosidic acid.

OH (No. 15 Shantou, Guangdong) was comprehensively analyzed by the UHPLC-MS method in the negative ion mode. Parent ion spectrums of the main ingredients are shown in Figure 2. Fragment ion spectrums (Supplementary Figure S5) of the main ingredients were obtained by the UHPLC-Triple-TOF/MS method. Based on the information of characteristic ions and fragment ions of primary and secondary mass spectrometry, the structure of the compounds was determined by matching through the mass spectrometry open source database such as Metlin, Reaxy, and MassBank. A total of 10 compounds were tentatively identified by inferring through mass spectrum fragment ion analysis or comparing with reference substances and literature data (Table 2) and chemical structures of them are shown in Figure 3. The MS spectrum of reference substances of ferulic acid detected by LCMS-2020 was shown in Supplementary Figure S5(J). These compounds included 5 iridoids, 2 anthraquinones, and 1 phenolic acid, and other 2 were unclassified and compound 3 and 4 were named by the IUPAC nomenclature as 6,7-dihydroxy-7-(hydroxymethyl)-1-(((2S,3R,4S,5S,6R)-3,4,5-trihydroxy-6-(hydroxymethyl)tetrahydro-2H-pyran-2-yl)oxy)-1,4a,7,7a-tetrahydrocyclopenta [c]pyran-4-carboxylic acid and 3-hydroxy-4-(((2S,3R,4S,5S,6R)-3,4,5-trihydroxy-6-((sulfooxy)methyl)tetrahydro-2H-pyran-2-yl)oxy)benzoic acid, respectively. The MS/MS spectra and the fragmentation pathway of asperulosidic acid are shown in Figure 4.

**TABLE 2:** Characterization of the chemical constituents of OH by using UHPLC-MS and UHPLC-Triple-TOF-MS.

Peak (no.)	*t*R (min.)	ESI-MS^-^	Mass (m/z)	Error (ppm)	Molecular formula	Fragment ions (m/z)	Identification	References
1	5	[M-H]^−^	389.1092	0.06	C16H22O11	89.0256, 119.0353, 137.0604, 165.0548,	Menotropin	Heffels et al. (2017)
						183.0649, 209.0442, and 227.0547		
2	7	[M-H]^−^	389.1098	0.06	C16H22O11	59.0176, 89.0258, 119.0359, 139.0389,	Deacetylasperulosidic acid	Zhao, X. et al. (2018)
						147.0445, 165.0551, 209.0447, and 227.0556		
3	7.9	[M-H]^−^	405.1047	0.00	C16H22O12	89.0249, 93.0356, 137.0603, 163.0392, 181.0500,	&	PubChem CID 2752072
						199.0604, 225.0392, 343.1034, and 361.1143		
4	8.6	[M-H]^−^	395.0297	0.00	C13H16O12S	79.9588, 96.9607, 109.0298, 138.9696, 153.0186,	#	Schidknecht (1986)
						166.9651, 241.0017, 315.0711, and 351.0392,		
5	10.4	[M-H]^−^	431.1213	0.00	C18H24O12	59.0179, 89.0256, 147.0444, 165.0550	Asperulosidic acid	Zhao, X. S. et al. (2018)
						225.0761, 251.0558, and 269.0661		
6	10.7	[M-H]^−^	389.1100	0.06	C16H22O11	69.0371, 89.0257, 121.0665, 139.0039,	Scandoside	Heffels et al., (2017)
						165.0555, 183.0656, 209.0438, and 345.1186		
7	11.5	[M + HCOO]^−^	459.1149	0.06	C18H22O11	59.0179, 119.0493, 147.0439,	Asperuloside	Zhao, X. S. et al. (2018)
						191.0334, 251.0557, and 413.1094		
8	13.1	[M + HCOO]^−^	653.2342	0.00	C28H32O15	283.0611	Hedanthroside C	Hu et al. (2011)
						and 253.0504		
9	13.7	[M-H]^−^	593.1558	0.00	C27H30O15	237.0548, 251.0344, 265.0506,	Hedanthroside B	Hu et al. (2011)
						269.0455, and 311.0560		
10	14.1	[M-H]^−^	193.0508	0.00	C10H10O4	134.0367, 178.0270, and 149.0606	Ferulic acid	Zhang et al. (2018)

& : 6,7-dihydroxy-7-(hydroxymethyl)-1-(((2S,3R,4S,5S,6R)-3,4,5-trihydroxy-6-(hydroxymethyl)tetrahydro-2H-pyran-2-yl)oxy)-1,4a,7,7a-tetrahydrocyclopenta [c]pyran-4-carboxylic acid.

# : **3**-hydroxy-4-(((2S,3R,4S,5S,6R)-3,4,5-trihydroxy-6-((sulfooxy)methyl)tetrahydro-2H-pyran-2-yl)oxy)benzoic acid.

Compound 1 produced predominant [M-H]¯ (at m/z 389.1092). Successive losses of CO_2_ and H_2_O molecule from fragment ion [M-H-Glc]¯ at m/z 227.0547 and [M-H-Glu]¯ at m/z 209.0442 produced another two minor ions at m/z 165.0548 and 147.0321. The fragment ion at m/z 183.0649 was probably formed by decarboxylation (–CO_2_) from the ion at m/z 227.0547. Further secessions of 44 and 18 Da (m/z 183.0649 and m/z 209.0442) can be attributed to the loss of CO_2_ and H_2_O. By searching the published literatures (Heffels et al., 2017), Monotropein was the most possible candidate for compound 1.

Compound 2 exhibited a predominant [M-H]¯ ion at m/z 389.1098. An obvious fragment ion [M-H-Glc]¯ at m/z 227.0556 was characterized by loss of a glucose residue (162 Da). Deacetylasperulosidic acid formed a product ion at m/z 209.0447 due to the loss of glucose (180 Da) from the deprotonated molecule [M-H]¯. The product ion at m/z 165.0511 was attributed to the loss of one molecule CO_2_ from m/z 209.0447 or one molecule CO_2_ and H_2_O from m/z 227.0556. The product ions at m/z were characterized by loss of one H_2_O from m/z 165.0511. This observation was consistent with the results from (Zhao X. et al., 2018) in which the MS data and the proposed fragmentation pathway of deacetylasperulosidic acid with molecular weight of 389.1098 Da have been reported. Therefore, deacetyl asperulosidic acid was considered to be suitable candidates for compound 2.

Compound 3 displayed a predominant [M-H]¯ ion at m/z 405.1047. The molecular formula is C_16_H_22_O_12_ and the fragment ions are at m/z 181.0500, 361.1143, 199.0539, 165.0511, 139.0389, 89.0528, and 227.0566.

Compound 4 exhibited a predominant [M-H]¯ ion at m/z 395.0297. The molecular formula was C_13_H_16_O_12_S and MS/MS fragmentation presented fragments are at m/z 153.0186, 241.0017, 109.0186, 96.9607, 351.0392, 315.0711, and 138.9696. Fragments at m/z 351.0392 were consistent with a decarboxylation from the ion at m/z 395.0297. The product ion at m/z 241.0017 was attributed to the loss of catechol (110Da) from m/z 351.0392. MS/MS fragmentation presented fragments at m/z 315.0711, 153.0186, and 109.0186 consistent with a desulfonation (315.0392) followed by a loss of a glucose moiety (153.0186) and further elimination of another CO_2_ (109.0186) molecule.

Compound 5 presented a [M-H]¯ ion at m/z 431.1213. The appearance of product ions at m/z 269.0661 and 251.0558 resulted from the loss of a glucose residue (Glc) (162 Da) and glucose (180 Da) from m/z 431.1213, respectively. The fragment ion at m/z 225.0761 was probably formed by decarboxylation (–CO_2_) from the ion [M-H-Glc]¯ at m/z 269.0661. The product ions at m/z 207.0342, 165.4334, and 147.0444 were formed by successive losses of H_2_O, COCH_3,_ and H_2_O from the fragment ion at m/z 225.0761. According to the literature (Zhao X. S. et al., 2018), compound 5 could be tentatively assigned as asperulosidic acid.

Compound 6 displayed a predominant [M-H]¯ ion at m/z 389.1100. Fragment ions at m/z 345.1186 and 209.0438 were formed by the losses of CO_2_ and glucose from the fragment at m/z 389.1100, respectively. Ion at m/z 183.0656 was attributed to the loss of glucose (180 Da) from m/z 345.1186. In addition, the fragment ions m/z 169.0555, m/z 121.0665, and m/z 139.0039 were occurred by the loss of CO_2_ (44 Da) and H_2_O (18 Da) from product ion at m/z 183.0656. Compound 6 could be reasonably assigned as scandoside (Heffels et al., 2017).

Compound 1, 2, and 6 were, respectively, monotropein, deacetylasperulosidic acid, and scandoside which were isomers of each other as they had a similar fragmentation pathway (Supplementary Figure S6). The main and typical losses of these compounds were H_2_O (18 Da), CO_2_ (44 Da), glucose residue (Glc) (162 Da), and glucose (180 Da). But the abundance of these fragment ions was different. In compound 2 and compound 6, the chiral structure of the carbon which was connected with the hex atomic ring and the hydroxy group were different in these two cases. Compound 2 showed an R type, while compound 6 was S type. With different chiral structure (S or R), there existed a stereo-selective reaction resulting in a different fragmentation pathway. And for compound 1, another isomer, with no hydroxy group in the carbon on the same position, would also lead to a different characteristic fragments. The compound with R type (compound 2) displayed priority losses of Glu and Glc residues from predominant [M-H]¯ ion at m/z 389.1098, following loss of one molecule CO_2_ from [M-H-Glu]¯ at m/z 209.0447 or [M-H-Glc]¯ at m/z 227.0556. While the compound with S type (compound 6) displayed priority losses of CO_2_ and Glu residues from predominant [M-H]¯ ion at m/z 389.1100, following loss of Glc residues from [M-H-COO]¯at m/z 345.1186. The compound without a hydroxy group on the same position (compound 1) displayed more similar fragmentation pathway to the compound with R type, without steric hindrance from hydroxy group. It displayed priority losses of Glu and Glc residues from predominant [M-H]¯ ion at m/z 389.1092, following loss of one molecule CO_2_ from [M-H-Glu]¯ at m/z 209.0442 or [M-H-Glc]¯ at m/z 227.0547.

Compound 7 (m/z 459.1149) was unambiguously tentatively identified as asperuloside (Zhao X. et al., 2018). The major ion observed in first-order mass spectra of compound 7 was a formic acid adduct (FAA) of its molecular ion, confirmed by loss of 46 Da in MS2. The fragment of asperuloside was obtained by a neutral loss of a glucose residue [M-H-162]¯. The product ions was observed at m/z 147.0439 [M-H-Glc-acetic acid (AA)-CO_2_]¯ together with a minor fragment at m/z 191.0334 [M-H-Glc-AA]¯. Consecutive loss of 28 Da led to formation of ion [M-H-Glc-AA-CO_2_-CO]¯ at m/z 119.0439.

Compound 8, at m/z 653.2342, corresponded to the formula [M + HCOO]¯. Its MS/MS spectrum showed a main fragment [M-H-324]¯ at 283.0611, which was consistent with the loss of methanoic acid and two glucose residues. According to the results of high resolution mass spectrometry, the molecular formula is C_28_H_32_O_15_. This compound was searched and speculated to be hedanthroside C through Scifinder and the PubChem database.

The MS/MS spectrum of compound 9 showed a molecular ion [M-H]¯ at m/z 593.1558 and fragments at m/z 269.0455, 251.0344, 265.0506, 237.0548, and 311.0560. Fragment 593.1558 corresponded to [M-H]¯, whereas ions at m/z 269.0455, 251.0344, and 237.0548 were consistent with the loss of two glucose residues followed by successive dehydration and demethylation, respectively. Compound 9 was speculated to be hedanthroside B through Scifinder and the PubChem database.

The appearance time of peak 10 is 14.1 min. Compound 10 was tentatively identified as ferulic acid by comparing the m/z of molecular ion [M-H]¯ and retention time between compound 10 and reference substance of ferulic acid Supplementary Figure S5(J).

### UHPLC Fingerprints Analysis

**FIGURE 5:**
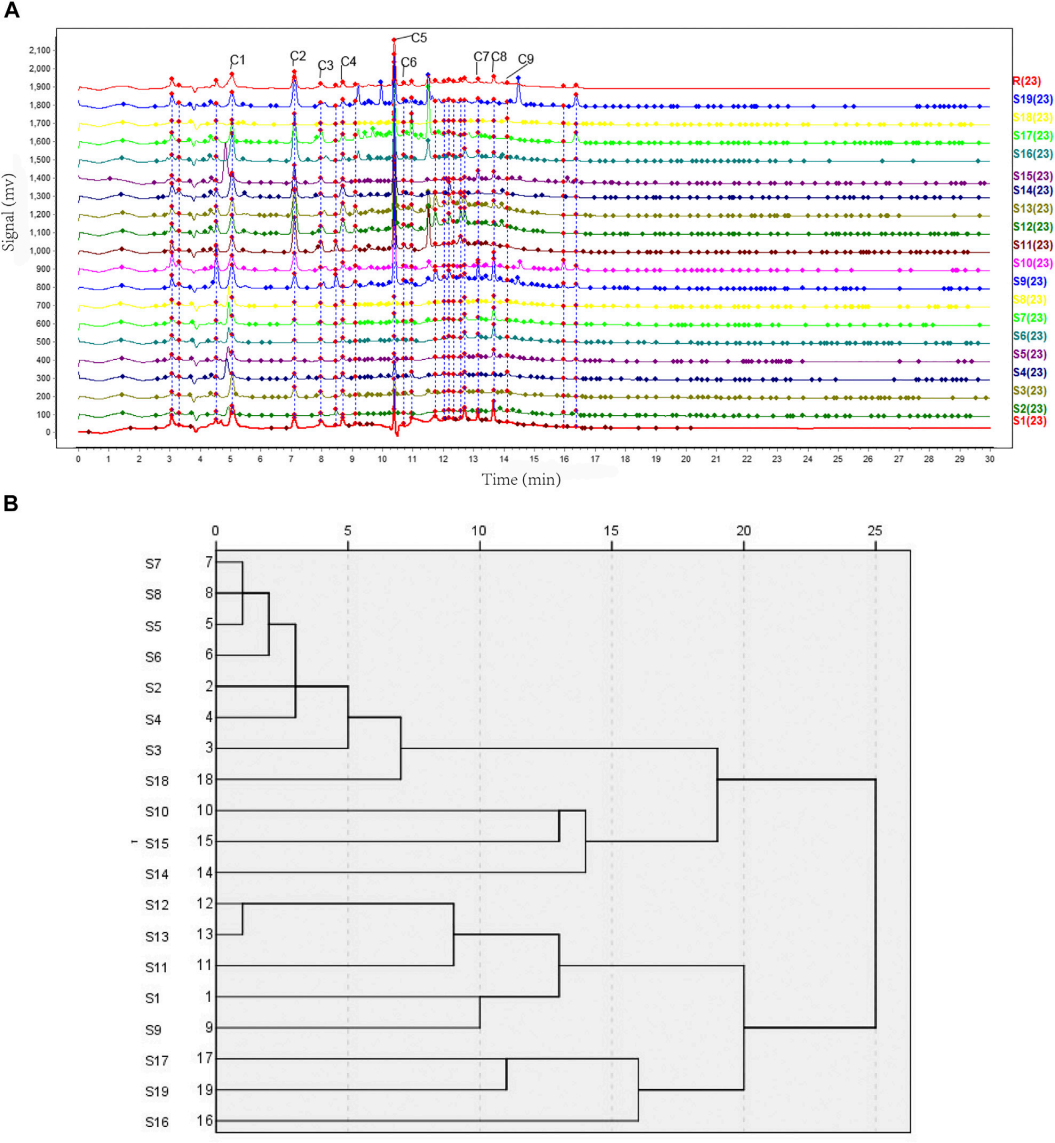
UHPLC fingerprints analysis and hierarchical cluster analysis. **(A)** The UHPLC fingerprints of 19 batches of OH samples (S1–S19) and reference standard fingerprint (R). C1—Monotropein. C2—Deacetylasperulosidic acid. C3—6,7-dihydroxy-7-(hydroxymethyl)-1-(((2S,3R,4S,5S,6R)-3,4,5-trihydroxy-6-(hydroxymethyl)tetrahydro-2H-pyran-2-yl)oxy)-1,4a,7,7a-tetrahydrocyclopenta [c]pyran-4-carboxylic acid. C4—3-hydroxy-4-(((2S,3R,4S,5S,6R)-3,4,5-trihydroxy-6-((sulfooxy)methyl)tetrahydro-2H-pyran-2-yl)oxy)benzoic acid. C5—Asperulosidic acid. C6—Scandoside. C7—Hedanthroside C. C8—Hedanthroside B. C9—Ferulic acid. **(B)** Clustering analysis graph of the 19 batches of OH.

We aimed to easily distinguish the quality of the OH from different regions and harvest dates. Nineteen batches (S1–S19) of OH were analyzed. The chromatogram of R was set as the standard fingerprint chromatogram, and a total of 23 common peaks were aligned and marked manually as common peaks, including the nine compounds tentatively identified in the UHPLC-MS and UHPLC-Triple-TOF-MS analysis. The chromatographic fingerprint of nineteen batches of samples was shown in (Figure 5A). Peak 5 (retention time = 10.4 min), a component with a consistently high concentration, was found commonly in 23 chromatograms. Therefore, this peak was used as the reference peak characteristic peaks.

### Similarity Analysis

The similarity of the 19 batches of samples are listed in Table 3. It shows that most samples had a similarity level of 0.892–0.937. The similarities of the fingerprint chromatograms of 19 samples compared to the standard fingerprint chromatogram were not more than 0.9, which indicated the differences of those 19 samples to a certain extent. The above analysis results further illustrated the influence of regional differences on the quality of OH and the regional differences had a greater influence than the harvest date on it.

**TABLE 3:** The similarity of 19 batches of samples.

No.	Similarity	Total area of common peaks (µv s)
S1	0.736	18250832
S2	0.847	2902125
S3	0.873	5115889
S4	0.849	4200118
S5	0.819	3337806
S6	0.86	4082344
S7	0.831	4277867
S8	0.841	3830185
S9	0.786	14397865
S10	0.771	6799104
S11	0.758	9775487
S12	0.842	13573788
S13	0.784	11592514
S14	0.792	10028262
S15	0.811	8800927
S16	0.817	8166084
S17	0.876	8932330
S18	0.763	3072093
S19	0.851	7379419

### Hierarchical Cluster Analysis

Based on the peak areas of the 23 aligned and marked manually compounds. The relative standard deviation (RSD) of the relative retention time of those 23 common peaks was less than 1% (see Supplementary Table S2, including area percentage of those 23 common peaks). The graph in (Figure 5B) illustrated that 19 batches (S1–S19) of OH could be classified into four groups at distance 15–20. S16, S17, and S19, named as group 1, were clustered together for the same character that the medicinal materials of them were branches and leaves, while others were stems. Group 2 contained S9, S11, S12, and S13 from Hezhou, Guangxi, and S1 from Shantou, Guangdong in China. Group 3 contained S10, S14, and S15, Group 4 contained S2, S3, S4, S5, S6, S7, S8, and S18 mainly from Yulin, Guangxi, which was considered as the worst group of the 19 samples related to analysis with the total peak area of 23 common peaks by the descending order. The total peak area of 23 common peaks of group 4 was the least, of group 3 and 1 were similar, of group 2 was the most. From the result, the quality of OH from Hezhou, Guangxi or Shantou, Guangdong, could be better, and as for medicinal parts, stems were better than branches and leaves. Cluster analysis also demonstrated that the medicinal materials of OH originating from the same district were not categorized together, which could be due to the variations in harvesting time, illumination intensity, planting patterns, and other factors.

### The Quantitative Analysis by UHPLC-UV

The developed UHPLC method was validated by assessing accuracy and precision. The recoveries of samples were found to be in the assortment of 98.12–106.97% with RSD ranging from 0.08 to 0.27% (Table 4). The RSD value of 0.079% was observed for precision (Table 5). The correlation coefficient (*r* ^2^) of asperulosidic acid (*r* ^2^ = 0.9990) showed a satisfactory linearity of the developed method (Table 6). These results showed that the developed method was precise and accurate for the quantitative estimation of asperulosidic acid in OH. The quantitative analysis of asperulosidic acid in the aqueous extract from the nineteen batches of samples was conducted by UHPLC-UV (Table 7 and Supplementary Table S2).

**TABLE 4:** Recovery for the analysis of the asperulosidic acid.

Original (μg)	Spiked (μg)	Detected (μg)	Average recovery (%)	RSD (%) (*n* = 3)
	21.11	65.23	106.97	0.08
42.77	41.86	85.54	101.84	0.16
	62.97	104.58	98.12	0.27

**TABLE 5:** Precision for the analysis of the asperulosidic acid (*n* = 6).

No.	Peak area (μv s)	μg/ml
1	772,990	42.75
2	773,390	42.77
3	774,110	42.81
4	773,941	42.8
5	772,942	42.75
6	772,416	42.72
Average (μg/ml)	773,298	42.77
RSD (%)		0.079

**TABLE 6:** Calibration curve for the analysis of the asperulosidic acid.

Regression equation (*γ* = ax + b)	*r* ^2^	Linear range (µg ml^−1^)
γ = 19,163x–46,219	0.9990	7.28–116.48

**TABLE 7:** The analysis of the asperulosidic acid from *Oldenlandia hedyotidea* (DC.) Hand.-Mazz.

No.	Content of asperulosidic acid from water extract from OH (μg/ml)	Content of asperulosidic acid from OH (mg/g)	RSD (%)
S1	55.30	1.11	1.19
S2	5.51	0.11	1.65
S3	22.77	0.45	1.56
S4	21.20	0.42	1.55
S5	7.99	0.16	2.64
S6	6.29	0.13	2.55
S7	13.51	0.27	2.59
S8	11.289	0.23	2.54
S9	148.20	2.96	0.53
S10	63.80	1.28	0.53
S11	130.03	2.60	0.72
S12	177.57	3.55	0.72
S13	149.34	2.99	0.83
S14	180.65	3.61	0.83
S15	42.77	0.85	1.68
S16	76.38	1.53	0.65
S17	95.26	1.90	0.65
S18	3.49	0.07	2.99
S19	68.99	1.38	0.58

### Anticancer Activity

**FIGURE 6:**
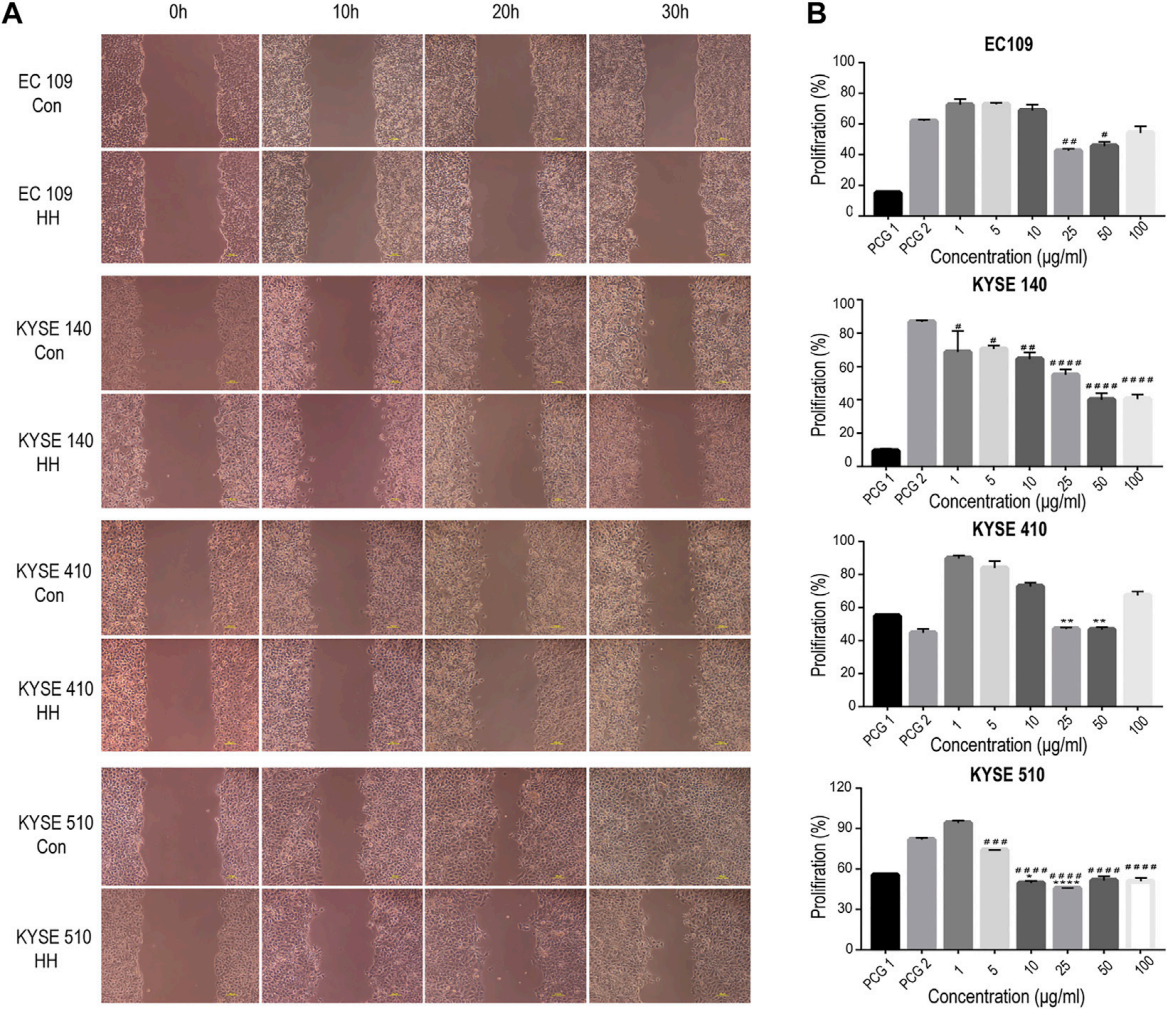
Water extract from OH inhibits migration and growth in the line of the esophageal cancer cell (EC109, KYSE140, KYSE410, and KYSE510). **(A)** The migration rate was measured by the wound healing assay. **(B)** Comparison of different doses of the water extract of OH on cell viability using the CCK-8 assay. Error bars represent SD (standard deviation), *indicates a difference compared with the PCG 1 group. *p < 0.1, **p < 0.01, ***p < 0.001, and ****p < 0.0001. ^#^ indicates a difference compared with the PCG 2 group. ^#^ *p* < 0.1, ^##^ *p* < 0.01, ^###^ *p* < 0.001, and ^####^ *p* < 0.0001.

The extract with higher concentration especially over 50 μg/ml had a significant inhibitory effect on cell activity (Figure 6A). The half maximal inhibitory concentrations of OH on the line of the esophageal cancer cells were between 32.81 and 104.9 μg/ml, not more than 105 μg/ml (Table 8). The cell inhibition ratio of esophageal cancer cells was significantly reduced by the extract of OH with a dose dependent effect under the effective IC50. Combining data from the four cell lines, we concluded that the most effective dose was 50 μg/ml.

**TABLE 8:** The half maximal inhibitory concentrations of OH on the line of esophageal cancer cells.

Type of esophageal cancer cell	IC50/μg/ml
EC109	55.79
KYSE140	32.81
KYSE410	104.9
KYSE510	41.64

The scratch assay showed that the cells in the control group moved to the scratch gradually after intervention of OH extract of IC50, while the cells in the treatment group moved slowly, cell morphology changed, and some of the cells were suspended and died in the later stage. Compared with the control group, with the prolongation of the drug action time, the cells in the treatment group did not heal or even the scratch area expanded (Figure 6B), and the scratch healing rate (Table 9) was much lower than that in the control group. It shows that the healing ability of tumor cells is significantly inhibited and the cell mobility is weakened under the effect of effective IC50 concentration and the extract can inhibit the activity and movement of tumor cells. The antitumor effect of OH can be achieved by inhibiting cell migration and promoting cell apoptosis.

**TABLE 9:** Scratch healing rate before and after administration.

Type of esophageal carcinoma cell	Group	Healing rate		
		10 h	20 h	30 h
EC109	Con	10.2	21.0	30.2
	HH	8.3	9.8	11.0
KYSE140	Con	25.2	37.8	43.9
	HH	18.5	28.8	29.8
KYSE410	Con	23.5	31.4	47.2
	HH	11.4	15.9	20.8
KYSE510	Con	43.2	65.7	100
	HH	31.6	33.9	48.3

It has been reported that OH has anti-inflammatory (He et al., 2012a) and hepatoprotective properties. However, it has not been reported whether it has anticancer activity or not. *Scleromitrion diffusum* (Willd.) R.J. Wang (SD) is also a member of the genus *Hedyotis*. It is known for its properties of heat-clearing and detoxification (in Chinese, Qing Re Jie Du), promotion of blood circulation and the removal of blood stasis (in Chinese, Huo Xue Hua Yu), and antitumor effect. It has been reported that SD has effects on liver cancer (Li et al., 2016), lung cancer (Su et al., 2019), breast cancer (Yang et al., 2020), prostate cancer (Song et al., 2019), gastric cancer (Liu et al., 2018a), and leukemia (Wang et al., 2011), especially in the aspect of digestive tract tumors, and has obvious effects on colorectal cancer (Sun et al., 2016; Liu et al., 2018b; Li et al., 2019). The antitumor activity of OH may be due to that it has some same chemical components with SD. The chemical constituents of SD mainly include flavonoids, iridoid glycosides, anthraquinones, and other compounds (Xu and Sung, 2005). According to our experiment, the main chemical components in the OH are also iridoids, anthraquinones, etc. Iridoid glycosides are a kind of vital compounds in natural products, and also one of the main chemicals in SD (Xu and Sung, 2005). There are asperuloside (Yang et al., 2014), monotropein, deacetylasperulosidic acid, scandoside, asperuloside acid (Wang et al., 2018), and ferulic acid in OH (Liang et al., 2008; Zhu et al., 2014; Zhai and Lv, 2016). Asperuloside exhibited evident cytotoxicity to HL-60, A459, HepG2, BGC-823, CNE-2, and HCT15, and the IC50 values are from 16.5 to 40.4 μM (Wang et al., 2018) and a novel antileukemic activity (Wang et al., 2011); it also shown an antitumor effect on mice with Lewis lung cancer (Chu et al., 2020). Asperulosidic acid showed moderate cytotoxicity to HL-60 and HepG2 (Wang et al., 2018). Monotropein had a potential therapeutic effect on colorectal cancer (Chong et al., 2020). Ferulic acid had the effects on the proliferation of human gastric cancer SGC-7901 cells (Niu et al., 2019) and it could also be used for the treatment of lung and liver cancer (Das et al., 2019; Rezaei et al., 2019). Tributyltin (IV) ferulate, a novel synthetic ferulic acid derivative, could induce autophagic cell death in colon cancer cells (Pellerito et al., 2020). Through the antitumor effects of these chemical components in OH, the antitumor effects of OH may through triggering autophagic (type II) cell death (Pellerito et al., 2020), promoting apoptosis (Wang et al., 2011), and attenuating the migration of cells and their tube formation abilities (Li et al., 2019). From the present study, it can be concluded that the results suggest that the use of OH may be beneficial for the treatment of esophageal cancer.

## Data Availability

The original contributions presented in the study are included in the article/Supplementary Material, further inquiries can be directed to the corresponding authors.
