# Supplementary figures and images for: The Analyses of Chemical Components From Oldenlandia hedyotidea (DC.) Hand.-Mazz and Anticancer Effects in vitro
Source: Front Pharmacol. 2021 May 10;12:624296. doi: 10.3389/fphar.2021.624296 (PMC8141642; doi:10.3389/fphar.2021.624296)

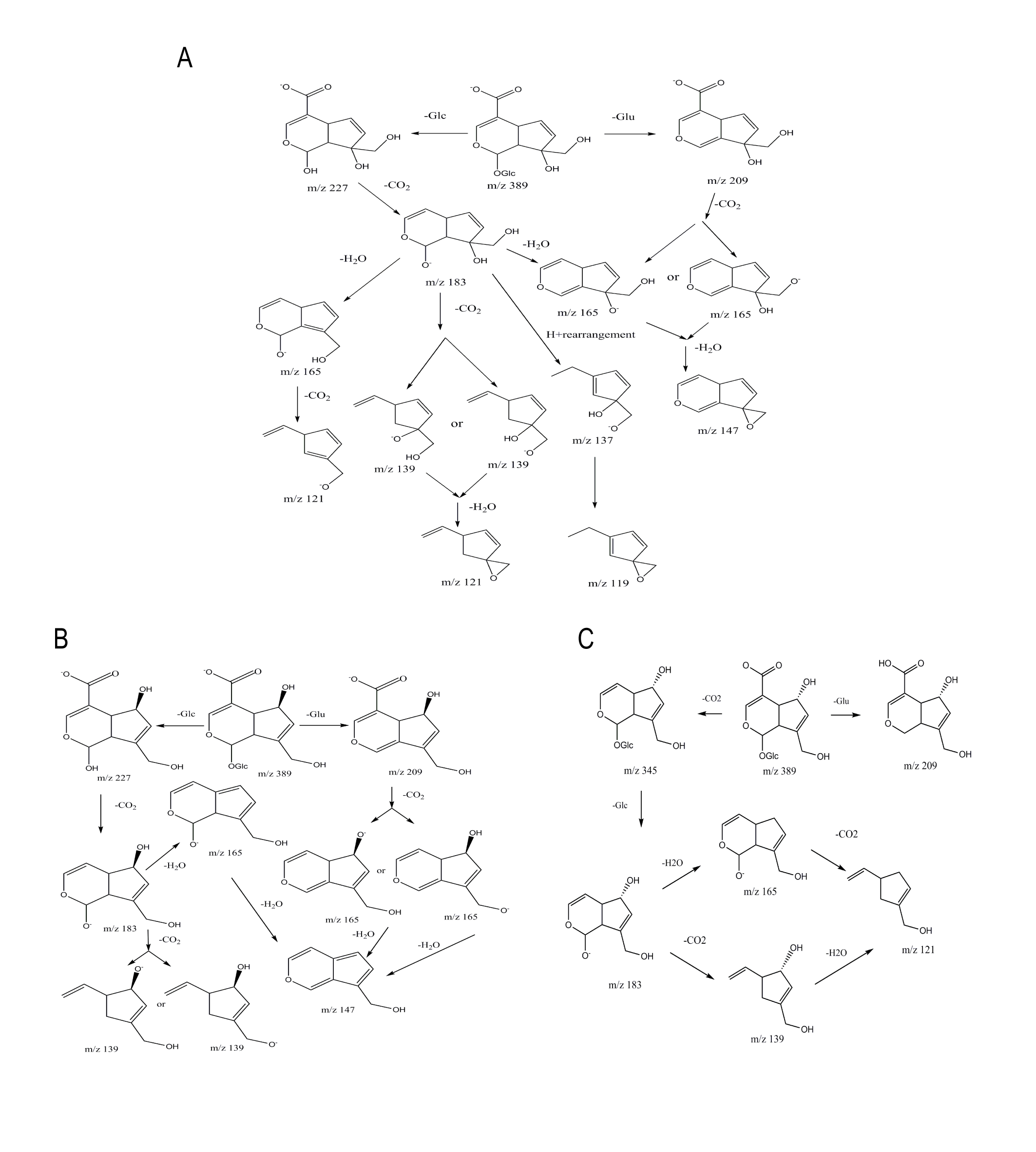

Supplement: Supplementary file 1 [file Image6.TIF]

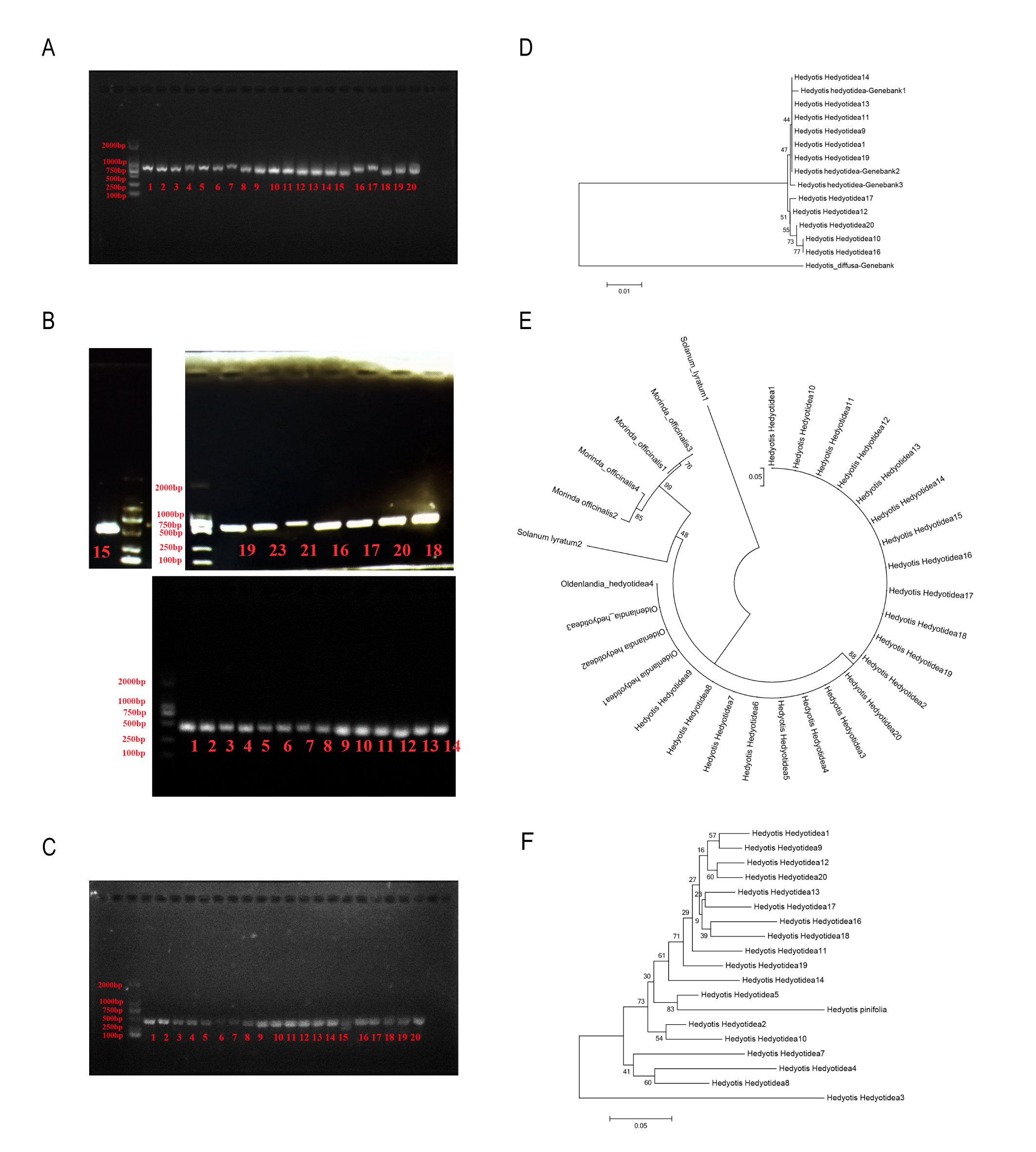

Supplement: Supplementary file 2 [file Image3.TIF]

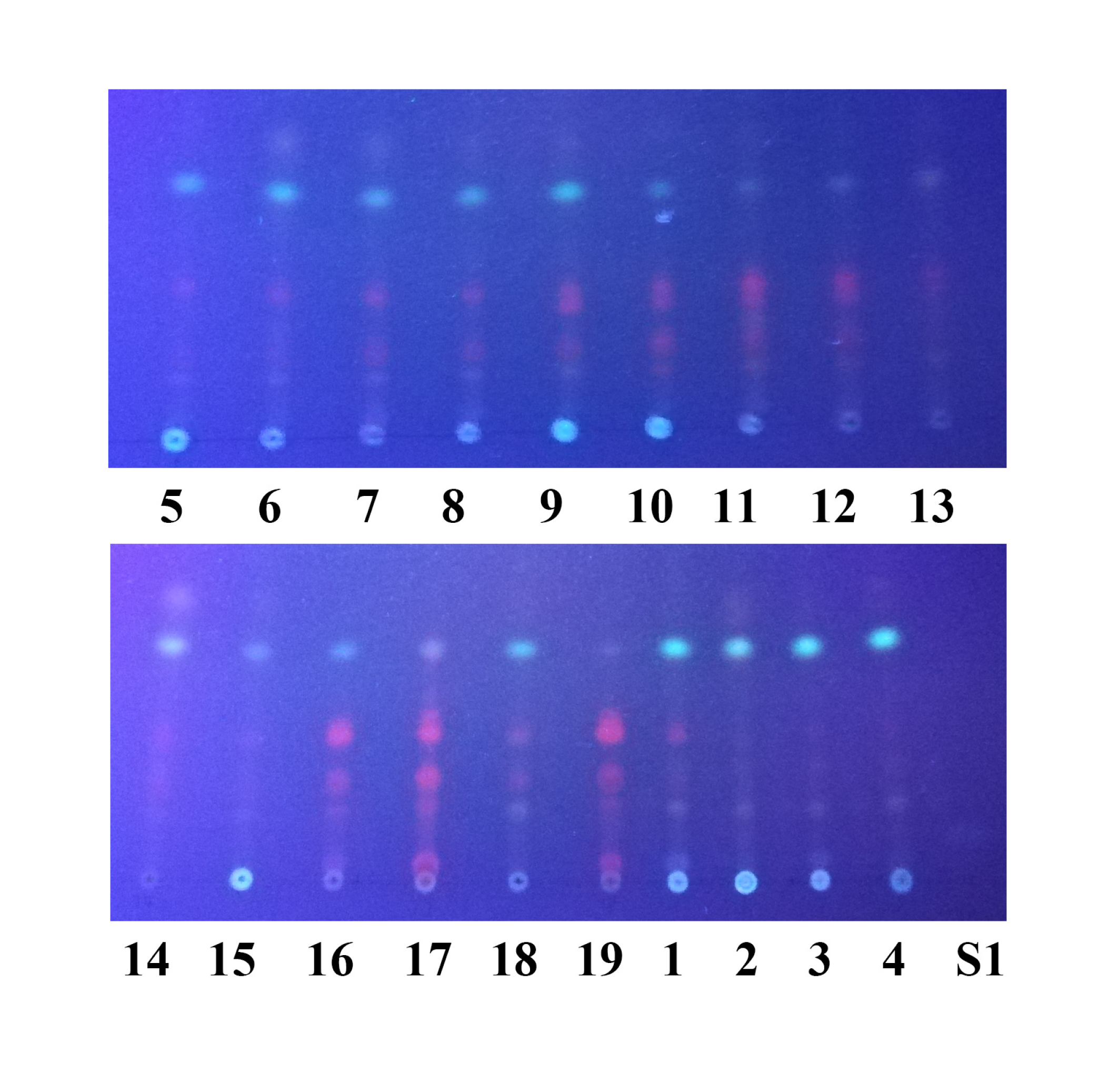

Supplement: Supplementary file 3 [file Image4.TIF]

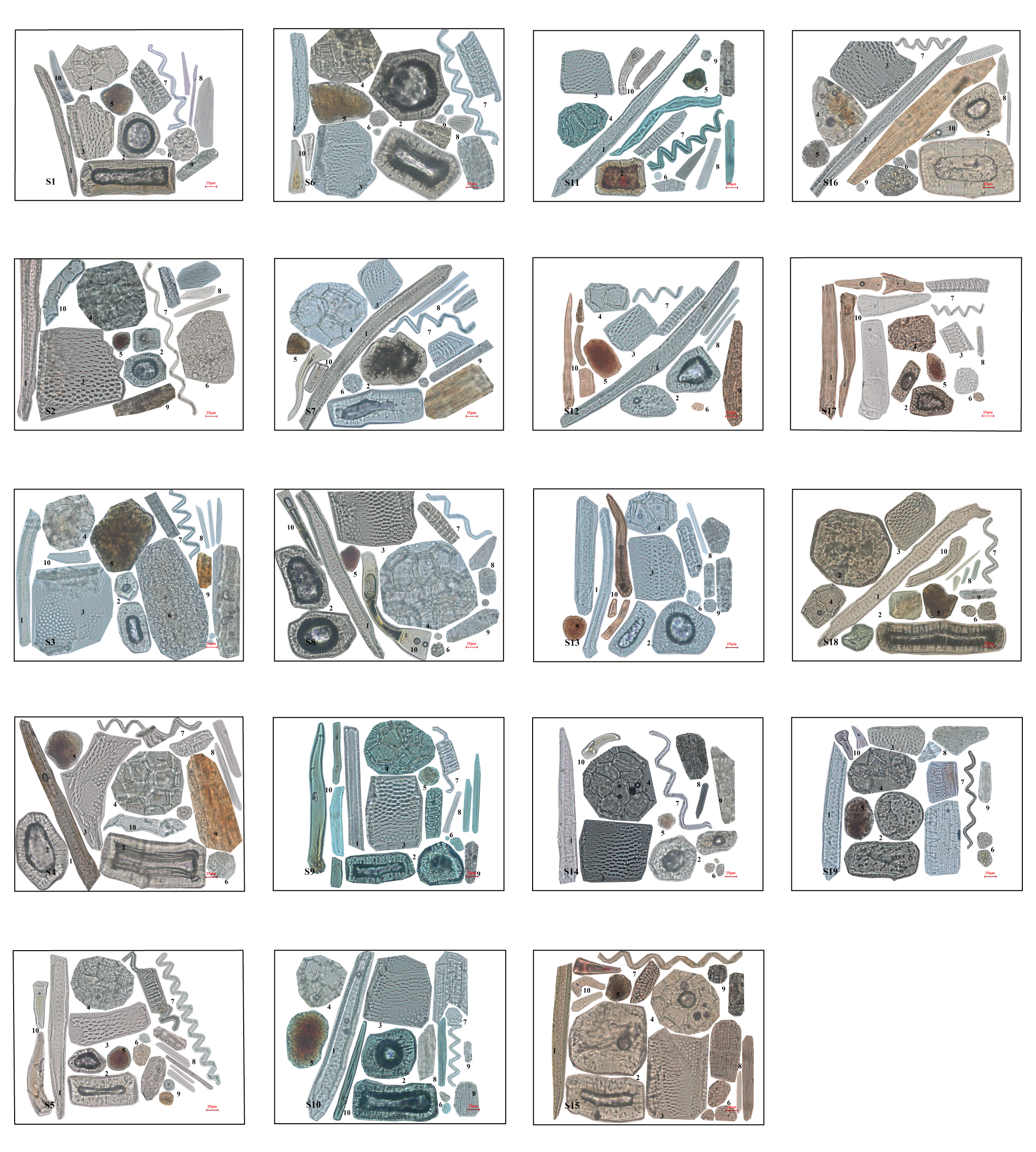

Supplement: Supplementary file 5 [file Image2.TIF]

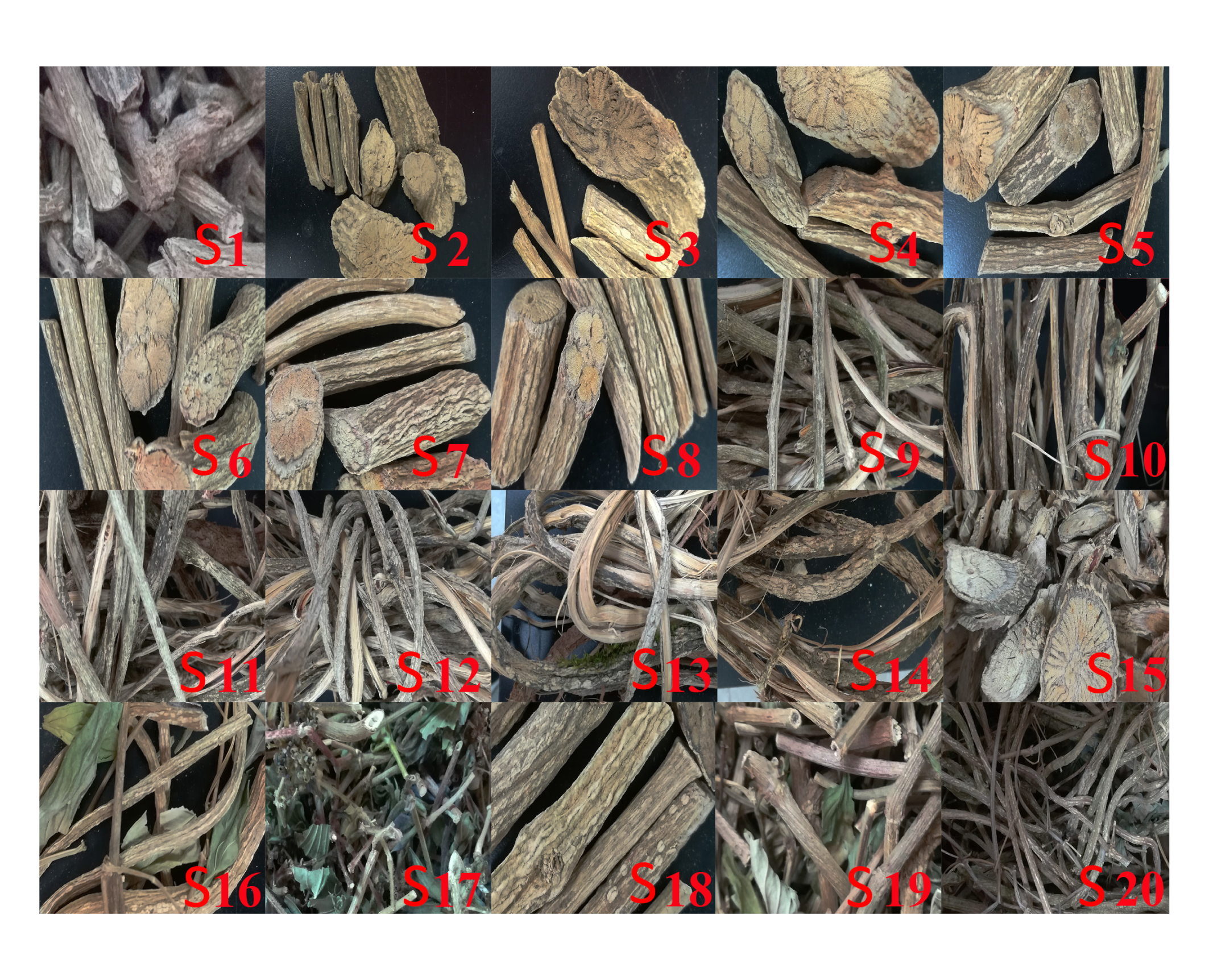

Supplement: Supplementary file 6 [file Image1.TIF]

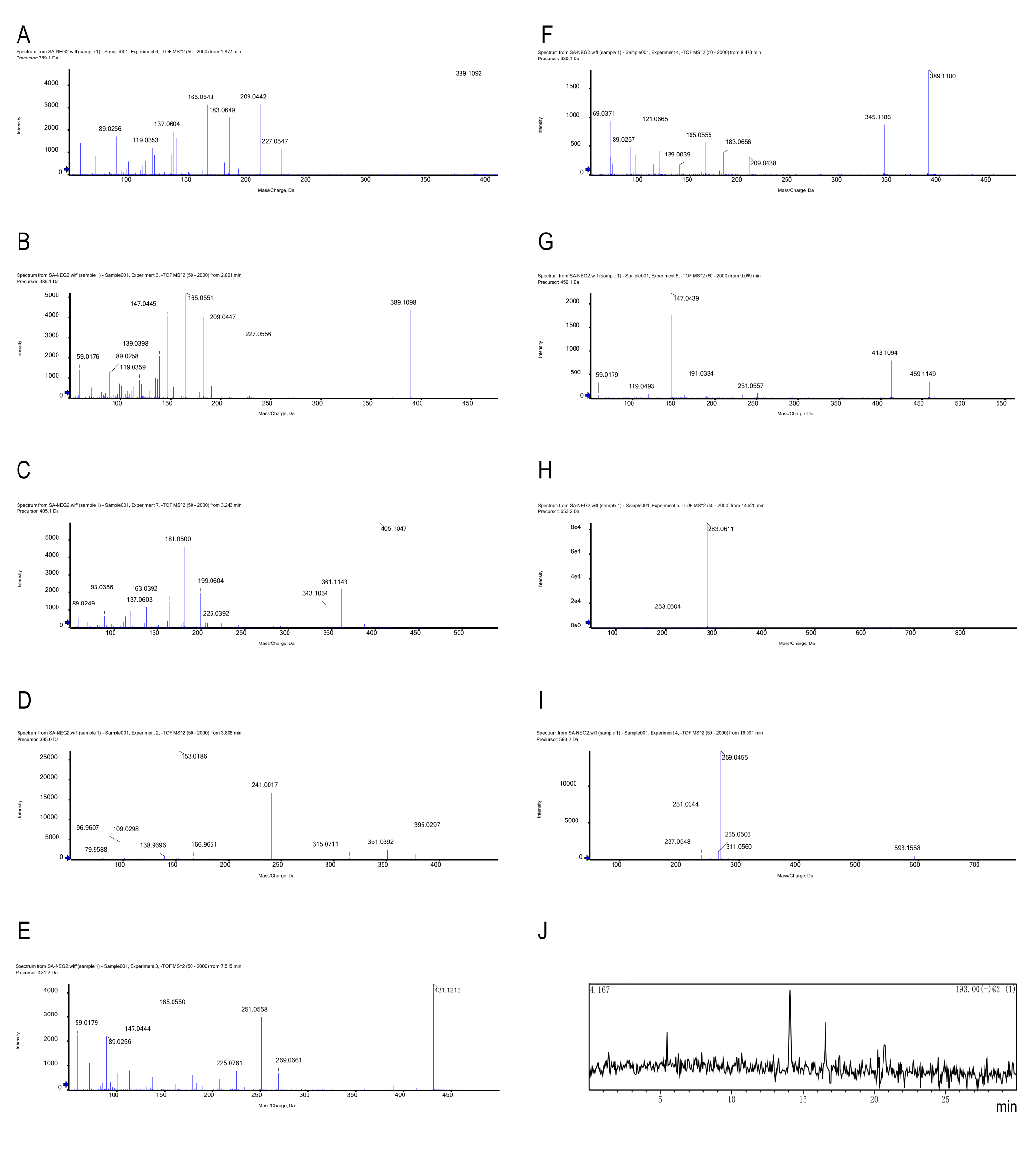

Supplement: Supplementary file 9 [file Image5.TIF]
