# Supplementary material for: The Analyses of Chemical Components From Oldenlandia hedyotidea (DC.) Hand.-Mazz and Anticancer Effects in vitro
Source: Front Pharmacol. 2021 May 10;12:624296. doi: 10.3389/fphar.2021.624296 (PMC8141642; doi:10.3389/fphar.2021.624296)
Supplement: Supplementary file 4 [file Table3.DOC]

TABLE S1 Origin of medicinal materials

| Number | Code | Sample name | Origin | Collection Time | Coordinates |
| --- | --- | --- | --- | --- | --- |
| 1 | S1 | Oldenlandia hedyotidea (DC.) Hand.-Mazz | Shantou,Gangdong | 20180926 | 116º 70’E, 23º 37’N |
| 2 | S2 | Oldenlandia hedyotidea (DC.) Hand.-Mazz | Chaozhou,Gangdong | 20180925 | 116º63’E, 23º66’N |
| 3 | S3 | Oldenlandia hedyotidea (DC.) Hand.-Mazz | Yulin,Guangxi | 20180927 | 110º14’E, 22º64’N |
| 4 | S4 | Oldenlandia hedyotidea (DC.) Hand.-Mazz | Lijiang,Yunnan | 20180927 | 100º25’E, 26º86’N |
| 5 | S5 | Oldenlandia hedyotidea (DC.) Hand.-Mazz | Yulin,Guangxi | 201806 | 110º14’E, 22º64’N |
| 6 | S6 | Oldenlandia hedyotidea (DC.) Hand.-Mazz | Yulin,Guangxi | 201807 | 110º14’E, 22º64’N |
| 7 | S7 | Oldenlandia hedyotidea (DC.) Hand.-Mazz | Yulin,Guangxi | 201808 | 110º14’E, 22º64’N |
| 8 | S8 | Oldenlandia hedyotidea (DC.) Hand.-Mazz | Yulin,Guangxi | 20180925 | 110º14’E, 22º64’N |
| 9 | S9 | Oldenlandia hedyotidea (DC.) Hand.-Mazz | Hezhou,Guangxi | 20180930 | 111º55’E, 24º41’N |
| 10 | S10 | Oldenlandia hedyotidea (DC.) Hand.-Mazz | Hezhou,Guangxi | 20180930 | 111º55’E, 24º41’N |
| 11 | S11 | Oldenlandia hedyotidea (DC.) Hand.-Mazz | Hezhou,Guangxi | 20180930 | 111º55’E, 24º41’N |
| 12 | S12 | Oldenlandia hedyotidea (DC.) Hand.-Mazz | Hezhou,Guangxi | 20180930 | 111º55’E, 24º41’N |
| 13 | S13 | Oldenlandia hedyotidea (DC.) Hand.-Mazz | Hezhou,Guangxi | 20180930 | 111º55’E, 24º41’N |
| 14 | S14 | Oldenlandia hedyotidea (DC.) Hand.-Mazz | Hezhou,Guangxi | 20180930 | 111º55’E, 24º41’N |
| 15 | S15 | Oldenlandia hedyotidea (DC.) Hand.-Mazz | Shantou,Guangdong | 20170902 | 116º 70’E, 23º 37’N |
| 16 | S16 | Oldenlandia hedyotidea (DC.) Hand.-Mazz | Maoming,Guangdong | 20171209 | 110º91’E, 21º65’N |
| 17 | S17 | Oldenlandia hedyotidea (DC.) Hand.-Mazz | Guigang,Guangxi | 20171213 | 109º60’E, 23º09’N |
| 18 | S18 | Oldenlandia hedyotidea (DC.) Hand.-Mazz | Yulin,Guangxi | 20171202 | 110º14’E, 22º64’N |
| 19 | S19 | Oldenlandia hedyotidea (DC.) Hand.-Mazz | Xiamen,Fujian | 20171202 | 118º11’E, 24º49’N |
| 20 | S20 | Oldenlandia hedyotidea (DC.) Hand.-Mazz | Hezhou,Guangxi | 20171210 | 111º55’E, 24º41’N |
| 21 |  | Gynochthodes officinalis (F.C.How) Razafim. & B.Bremer | Chaozhou,Gangdong | 20171209 | 116º63’E, 23º66’N |
| 22 |  | Gynochthodes officinalis (F.C.How) Razafim. & B.Bremer | Jieyang,Guangdong | 20171221 | 116º35’E, 23º54’N |
| 23 |  | Solanum lyratum Thunb. | Bozhou,Anhui | 20171209 | 115º78’E, 33º86’N |
