# Supplementary material for: The Analyses of Chemical Components From Oldenlandia hedyotidea (DC.) Hand.-Mazz and Anticancer Effects in vitro
Source: Front Pharmacol. 2021 May 10;12:624296. doi: 10.3389/fphar.2021.624296 (PMC8141642; doi:10.3389/fphar.2021.624296)
Supplement: Supplementary file 7 [file Table1.DOC]

**FIGURE S1.** Picture of medicinal materials of Oldenlandia hedyotidea (DC.) Hand.-Mazz(OH) . Origin of S1-S20 of OH were consistent with Table S1.

**FIGURE S2.** Microscopic identification of OH. **1,** tracheids **2,** stone cells **3,** bordered pitted vessels **4,** cork cells **5,** oil cells **6,** starch granules **7,** spiral vessels **8,** raphides of calcium oxalates **9,** crystalline sheath fibers (from which we can see clusters of calcium oxalate on the surface) **10,** nonglan-dular hairs.

**FIGURE S3.** Picture of plant DNA Barcode technology. Efficiency of DNA extraction and PCR amplification were shown on electrophoregram ITS (A), ITS2 (B) and psbA-trnH (C). The NJ tree of ITS sequence was displayed that the ratio of genetic distance to branch length was 0.005/cm. 11 batches of sequences identified by ITS sequence as *Oldenlandia hedyotidea* (DC.) Hand.-Mazz genebank also known as *Hedyotis hedyotidea* could be clustered into the same clade as the 3 sequences downloaded by genebank (*Hedyotis hedyotidea* genebank 1-3), while ITS sequences of *Scleromitrion diffusum* (Willd.) R.J.Wang also named *Hedyotis diffusa Willd* downloaded by genebank could be clustered into different clade with large genetic distance. The results showed that ITS sequence could distinguish *Hedyotis hedyotidea* from other species in the same genus (D). The NJ tree of ITS2 sequence was shown that branch length represented the genetic distance between different sequences. The ratio of genetic distance to branch length was 0.05/cm. The ITS2 sequence of *Hedyotis hedyotidea* 1-20 and *Hedyotis hedyotidea* 1-4 downloaded by genebank were clustered together. The ITS2 sequence of *Gynochthodes officinalis* (F.C.How) Razafim. & B.Bremer also known as *Morinda Officinalis* 1,3 (Number 21-22) and *Morinda Officinalis* 2,4 downloaded by genebank were clustered together. The ITS2 sequence of *Solanum lyratum Thunb* (*Solanum Lyratum*) 1 (Number 23) was aggregated into another one. The ITS2 sequence of SLT 2 downloaded by genebank and *Morinda Officinalis* 1-4 were clustered together. Results showed that *Hedyotis hedyotidea* formed into one clade, which could be successfully distinguished from its adulterants. ITS2 sequence could be used as the DNA barcode of *Hedyotis hedyotidea* identification (E). The NJ tree of psbA-trnH sequence was exhibited that we could identify the majority of *Hedyotis hedyotidea* (Number 1-5,7-14,16-20) through psbA-trnH sequence, but the psbA-trnH sequence of all samples of *Hedyotis hedyotidea* could not be clustered into one branch. psbA-trnH sequence could not distinguish *Hedyotis hedyotidea* and *Scleromitrion pinifolium* (Wall. ex G.Don) R.J.Wang also known as *Hedyotis pinifolia* which from other species of the same genus (F). The results of NJ tree showed that ITS2 sequence could distinguish *Hedyotis hedyotidea* from its adulterants, ITS sequence could distinguish *Hedyotis hedyotidea* from other species of the same genus, but psbA-trnH sequence could not distinguish *Hedyotis hedyotidea* from other species of the same genus. So ITS2 sequence was the preferred sequence for identification of *Hedyotis hedyotidea.*

**FIGURE S4.** TLC chromatogram of Number 1-19 sample examined under 365 nm UV light and S1 was the reference substance of asperulosidic acid. We could find 4 spots.

**FIGURE S5.** Fragment ion spectrums of the main ingredients. **(A)** Monotropein. **(B)** Deacetylasperulosidic acid. **(C)** 6,7-dihydroxy-7-(hydroxymethyl)-1-(((2S,3R,4S,5S,6R)-3,4,5-trihydroxy-6-(hydroxymethyl)tetrahydro-2H-pyran-2-yl)oxy)-1,4a,7,7a-tetrahydrocyclopenta[c]pyran-4-carboxylic acid. **(D)** 3-hydroxy-4-(((2S,3R,4S,5S,6R)-3,4,5-trihydroxy-6-((sulfooxy)methyl)tetrahydro-2H-pyran-2-yl)oxy)benzoic acid. **(E)** Asperulosidic acid. **(F)** Scandoside. **(G)** Asperuloside. **(H)** Hedanthroside C. **(I)** Hedanthroside B. **(J)** The MS spectrum of reference substances of ferulic acid detected by LC/MS-2020.
