# Supplementary material for: The Analyses of Chemical Components From Oldenlandia hedyotidea (DC.) Hand.-Mazz and Anticancer Effects in vitro
Source: Front Pharmacol. 2021 May 10;12:624296. doi: 10.3389/fphar.2021.624296 (PMC8141642; doi:10.3389/fphar.2021.624296)
Supplement: Supplementary file 10 [file Table2.DOC]

**TABLE S1.** Origin of S1-S20 of samples

**TABLE S2.** The relative standard deviation (RSD) of the relative retention time of 23 common peaks and peak area.
